# Laser-Assisted Synthesis and Oxygen Generation of Nickel Nanoparticles

**DOI:** 10.3390/ma13184068

**Published:** 2020-09-13

**Authors:** Jakub Wawrzyniak, Jakub Karczewski, Jacek Ryl, Katarzyna Grochowska, Katarzyna Siuzdak

**Affiliations:** 1 Centre for Plasma and Laser Engineering, The Szewalski Institute of Fluid-Flow Machinery, Polish Academy of Sciences, Fiszera 14 st., 80-231 Gdańsk, Poland; kgrochowska@imp.gda.pl (K.G.); ksiuzdak@imp.gda.pl (K.S.); 2 Faculty of Applied Physics and Mathematics, Gdańsk University of Technology, Gabriela Narutowicza 11/12 st., 80-233 Gdańsk, Poland; jakub.karczewski@pg.edu.pl; 3 Faculty of Chemistry, Gdańsk University of Technology, Gabriela Narutowicza 11/12 st., 80-233 Gdańsk, Poland; jacek.ryl@pg.edu.pl

**Keywords:** nickel nanoparticles, pulsed laser ablation, oxygen evolution reaction, electrochemical performance

## Abstract

Nowadays, more than ever, environmental awareness is being taken into account when it comes to the design of novel materials. Herein, the pathway to the creation of a colloid of spherical, almost purely metallic nickel nanoparticles (NPs) through pulsed laser ablation in ethanol is presented. A complex description of the colloid is provided through UV-vis spectroscopy and dynamic light scattering analysis, ensuring insight into laser-induced nanoparticle homogenization and size-control of the NPs. The transmission electron spectroscopy revealed spherical nanoparticles with a narrow size distribution, whereas the energy-dispersive X-ray spectroscopy accompanied by the X-ray photoelectron spectroscopy revealed their metallic nature. Furthermore, an example of the application of the colloidal nanoparticles is presented, where a quick, five-min ultrasound modification results in over an order of magnitude higher current densities in the titania-based electrode for the oxygen evolution reaction.

## 1. Introduction

The interest in metal nanoparticles (NPs) is at an all-time high, due to them being able to perform in a plethora of applications, such as sensing [1], catalysis [2], antibacterial devices [3], and many more [4,5,6,7]. They are often synthesized using laborious, chemical methods leaving behind a trail of waste and byproducts. With growing concerns for the natural environment, other synthesis strategies are being developed, where the use of harmful compounds is minimized, simultaneously retaining high-purity products. One of such methods is pulsed laser ablation in liquid (PLAL), where only the metal plate and transparent solvent are used [8]. In the PLAL technique, the irradiation of solid target by laser causes the formation of plasma plume at solid-liquid interface, which contains neutral atoms, ions and electrons from the target. Next, plasma is adiabatically expanded and created shock wave increases the temperature and pressure inside the plasma. The vapor layer is formed due to the expansion and condensation of plasma as its energy is transferred to the solution. The vapor layer grows, forming the cavitation bubble and during the bubble expansion its pressure decreases as well as the temperature, and the solid crystallization occurs leading to the formation of nanoparticles. In the last stage, the bubble collapses and the nanoparticles are released into the liquid. The thorough description of nanoparticle creation by pulsed laser ablation in solution can be found in the work of Huang et al. [9]. In this process, the pulsed, nanosecond lasers are most commonly employed, and laser wavelength and fluence determine the size of the obtained NPs. For the highest efficiency, the laser beam should be focused slightly behind the target surface. Additionally, to prevent consecutive laser hits onto the same area and the creation of deep cavities, often slow rotation of the target is employed [10,11]. When oxidized nanoparticles are desired, frequently water is used as a medium, whereas organic solvents such as acetone or ethanol are common for metallic nanoparticles [12,13]. As stability is a concern when it comes to the usability of such colloids, the use of surfactants is quite common, such as cetrimonium bromide (CTAB) [14,15]. Unfortunately, these additives will inevitably affect the chemical properties of the obtained nanoparticles, which in certain applications is undesirable. Finally, PLAL allows for immediate dispersion of the nanoparticles in the solution, where they can be easily transported and used whenever needed. The most commonly created nanomaterials are the noble metal nanostructures. For example, silver nanoparticles are used in the food industry as an antibacterial agent and in sensing, enhancing Raman signal in SERS (Surface-enhanced Raman Spectroscopy) [16,17]. Gold nanomaterials are on the other hand the most prevalent materials in cancer research and drug delivery [18,19]. Whereas platinum is irreplaceable as a catalyst and is often applied toward effective hydrogen evolution reaction [20]. The abovementioned materials, however, have one thing in common, they are quite scarce and expensive. Therefore, lots of research towards the use of non-noble metals as a cheaper alternative is being conducted. The nickel nanostructures, especially, draw much attention. It has been found that nickel nanomaterials can exhibit unprecedented compressive strength [21], have pesticidal properties [22], perform in fuel cells [23], or can be used as an effective catalyst [24]. Moreover, the versatility of nickel allows it to be synthesized in various forms, such as foam, wires, spheres, shells, or tubes [25,26,27,28]. There are numerous works in which PLAL technique was introduced to obtain nickel nanoparticles. For example, Nikov et al. [29] studied nanosecond laser ablation of Ni in distilled water and ethanol. The authors observed the presence of bimodal size distribution, namely formation of nano- and microparticles. Interestingly, in the microscopic level, the NPs obtained in ethanol are bigger, while in nanoscale level the selected liquid did not influence the NP dimensions significantly. Moreover, three different wavelengths (355, 532 and 1064 nm) were used and the increase in average diameter was observed with the increased laser wavelength. Additionally, the authors showed that applying magnetic field indicates the possibility of controlled particle separation, depending on their size. The morphology of NiNPs obtained via PLAL technique was also reported by Torrisi et al. [30]. The NPs were produced for 20 min using the 1064 nm wavelength and it was shown that the concentration decreases with the increased fluence and process duration as the laser light is absorbed in the solution, leading to the partial fragmentation of NPs and hence the reduction in mean size distribution. The optical and morphological properties of NiNPs formed during laser irradiation by applying 532 and 1065 nm wavelength were also studied by Tan et al. [31]. It was found that the intensity of absorption peak was dependent on the laser wavelength and the laser pulse energy, while the size of NPs remains constant regardless of the chosen wavelength. The effects of laser fluence onto nanoparticle dimensions were investigated by Safa et al. [32] and it was proven that the size of NiNPs is decreasing with the increasing fluence of laser pulse.

Due to Ni flexibility and the wide range of possible applications, we wanted to present guidelines for the creation of metallic, spherical, Ni nanoparticles with a narrow size distribution, as well as characterize their key physical parameters. Moreover, we provide an example of modification with created colloid, using it to enhance the efficiency of oxygen production in the TiO_2_-based electrode by over an order of magnitude. Due to the easily adjustable TiO_2_ nanotubes (NTs) morphology, facile production, high surface area, and stability [33,34,35,36,37,38,39], together with nickel nanoparticles, they have become a very strong, yet eco-friendly, duet for further research. The electrochemical properties of hybrids composed of Ni nanoparticles electrodeposited onto titania nanotubes were shown for example by Chen et al. using nickel sulphate as NPs precursor [40]. It was reported that the size and amount of nickel nanoparticles have a huge impact onto the light absorption and therefore onto the light-to-heat conversion and energy harvesting. Such material also exhibits electrocatalytic activity towards methanol oxidation [41] and improved visible light photocatalytic activity through the introduction of impurity bands below the conduction band of titania [42]. Typically, reports show that titania NTs can be decorated by NiNPs via wet-impregnation [43], sputtering followed by dealloying [44], ultrasound deposition from NiCl_2_ [45] or electrodeposition realized under the constant current [40]. Nevertheless, in the vast majority of cases, additional thermal annealing above 300 °C is applied to decompose nickel precursor, which provides a stable integration with titania support or ensures crystallization of Ni particles. Herein, only five-min long contact of TiO_2_ with Ni colloid, followed by short drying, is sufficient to obtain the hybrid structure composed of NTs and NPs. Indeed, despite no changes in morphology, the change to the electrochemical activity was found and over an order of magnitude enhancement of current densities in oxygen evolution reaction was observed. We, therefore, present an analysis of not only NiNPs created with pulsed laser ablation in liquid but the decorated titania-based electrode as well.

## 2. Materials and Methods

The titania nanotubes were synthesized by electrochemical oxidation of the titanium foil in the two-electrode system. First, the titanium sheet (0.127 mm × 35 mm × 25 mm, 99.7% pure, Strem) was ultrasonically cleaned for 10 min in acetone, ethanol, and deionized (DI) water. After drying in air, the foil was immersed in an electrolyte based on diethylene-glycol, containing 0.3 wt % NH_4_F, 0.5 wt % HF, and 7 wt % DI water where it underwent anodization process at a thermostat-controlled temperature of 40 °C. The voltage was controlled by in-house build hardware with the ramp-up rate set to 0.1 V/s up to 30 V, where it was kept for two hours after which it was gradually lowered at the same rate. Afterward, the samples were rinsed with ethanol and dried in air. To obtain the crystalline anatase phase, the nanotubes were calcined in a furnace (Nabertherm, Lilienthal, Germany) at 450 °C for two hours. The 1064 nm pulsed Nd:YAG laser (6 ns, Quantel, Lannion, France) was used to synthesize nickel nanoparticles via pulsed laser ablation in liquid. The nickel plate (1 mm × 15 mm × 15 mm, 99.5%, HMW Hauer GMBH & Co., Rottenbach, Germany) was ultrasonically cleaned for 5 min in acetone and ethanol, and placed into a 20 mL beaker, which was given slow rotation by electric rotator to reduce the impact of consecutive laser pulses. The glass vessel was filled with 5.4 mL of ethanol and the laser spot size was ca. 1 mm^2^. The pulse fluence was set to 14.2 J/cm^2^ and the number of pulses was adjusted between 10 and 40 thousand via the laser control panel. Afterwards, 0.7 mL of the nanoparticle-rich solution was transferred to Eppendorf tubes along with NT-covered foil and placed in an ultrasonic bath for 5 min. The samples were then dried on a hot-plate at 40 °C for 5 min.

The absorbance spectra of the Ni colloid and Ni-decorated titania were obtained via the PerkinElmer dual-beam spectrophotometer (Lambda35, PerkinElmer, Waltham, MA, USA) at a scanning speed of 120 nm/min, while the X-ray diffraction (XRD) patterns were recorded using Bruker D2Phaser diffractometer (Bruker, Billerica, MA, USA) with Cu Kα radiation. The X-ray photoelectron spectroscopy (XPS) studies were carried out for Ni nanoparticles obtained at 40 thousand pulses and deposited onto Si substrate and titania support using Escalab 250Xi spectroscope (Thermo Fisher, Waltham, MA, USA) equipped in Al Kα monochromatic X-ray source, operating at 20 eV pass energy. Charge compensation was provided utilizing a flood gun, with the final calibration based on the signal derived from adventitious carbon (C1s at 284.6 eV). Images of nanoparticles were provided by transmission (TEM, JEOL ARM 200F equipped with energy-dispersive X-ray spectrometer - EDX, JEOL Ltd., Tokyo, Japan) and scanning electron (FE-SEM FEI Quanta FEG 250, Thermo Fisher, Waltham, MA, USA) microscopes, while their size and zeta potentials were determined by dynamic light scattering device (DLS) Anton Paar Litesizer 500 (Anton Paar, Gratz, Austria).

The electrochemical properties were studied in 0.5 M NaOH (deaerated with Ar) using Autolab PGStat 302N (Autolab, Utrecht, Holland) in a three-electrode system. Prepared samples acted as a working electrode, the platinum mesh was used as a counter electrode, and Ag/AgCl/0.1M KCl as a reference electrode. For UV-vis illumination, the AM 1.5 simulator (Oriel LS0500, LOT-Quantum Design GmbH, Darmstadt, Germany) was used. Before each measurement, cyclic voltammetry sweeps were performed to reach the chemical equilibrium. The linear voltammetric investigations were carried out from −1 to +1 V, with a sweeping speed of 10 mV/s. The electrochemical impedance spectra (EIS) were recorded in open circuit conditions in the dark and under UV-vis irradiation. The spectra were registered in the frequency range from 20 kHz to 0.1 Hz with 10 mV amplitude and with 10 point per decade providing 49 points. Before spectra recording, the electrode was conditioned for 10 min

## 3. Results and Discussion

### 3.1. Colloid Characterisation

**Figure 1:**
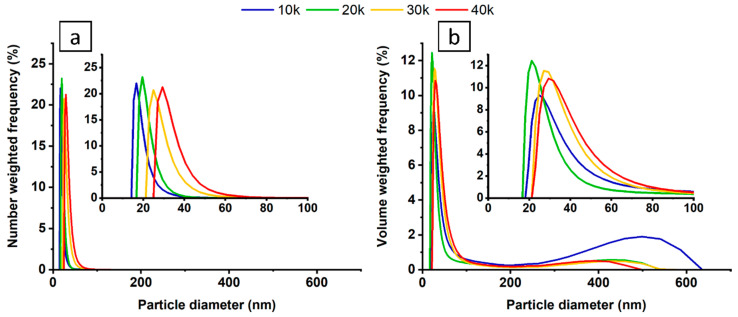
Relative frequency number (**a**) and volume (**b**) weighted by the nanoparticle size distribution for colloids varying in the number of applied laser pulses.

The analysis of the zeta potential and size distribution of the nanoparticles was investigated via dynamic light scattering technique, and the appropriate results are presented in Figure 1.

The zeta potential for the as-prepared colloids was determined at 29 ± 3 mV, which indicates that the nanoparticles are stable in the solution [46]. Figure 1a shows the NP hydrodynamic size distribution by their number and indicates that shorter irradiation time allows the creation of smaller nanoparticles (ca. 16 nm at 10 thousand pulses). Over time, however, only nanoparticles bigger than 25 nm are present. This is due to the laser-induced heating of the solution, which substantially lowers zeta potential during modification, increases Brownian motion, and therefore promotes agglomeration of the nanoparticles [10,46,47]. Figure 1b shows the volume-weighted size distribution of the particles in the colloid. Despite the low number of big particles (~500 nm), they visibly contribute to the overall volume in the colloid. Their presence might be explained by the detachment of the bigger chunks of the Ni plate into the solution when ablation was first initiated. Nonetheless, during the process they were split into smaller pieces, which confirms that laser irradiation might be used for homogenization of the nanoparticle solutions, or breaking-down aged solutions in which the nanoparticles have already agglomerated [29,48,49].

**Figure 2:**
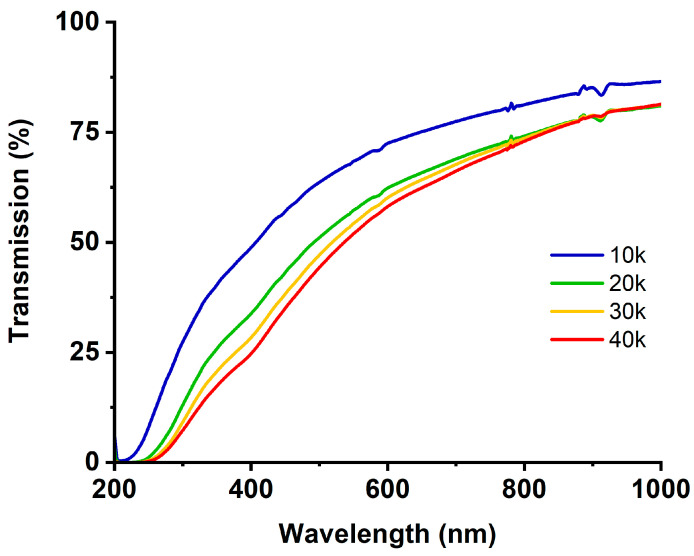
Transmission spectra obtained from pulsed laser ablation in liquid (PLAL) synthesis of nickel nanoparticles for 10–40 thousand laser pulses.

The optical transmission spectra are shown in Figure 2.

As could be expected, the absorbance of the investigated solution rises with the number of pulses and therefore the number of nanoparticles present. In all investigated cases, the absorbance rises with photon energies nearing total absorption at ca. 210 nm for synthesis with 10 thousand laser pulses and 260 nm for 40 k. Moreover, a slight absorption band is present at ca 370 nm, which gets more intensive with the number of pulses used and can be assigned to oxidized nickel [50,51]. Interestingly though, literature regarding the absorbance spectra of nickel particles is very inconsistent as some researchers report no absorption bands in the spectrum [52,53], while others claim multiple bands are present [54,55,56].

**Figure 3:**
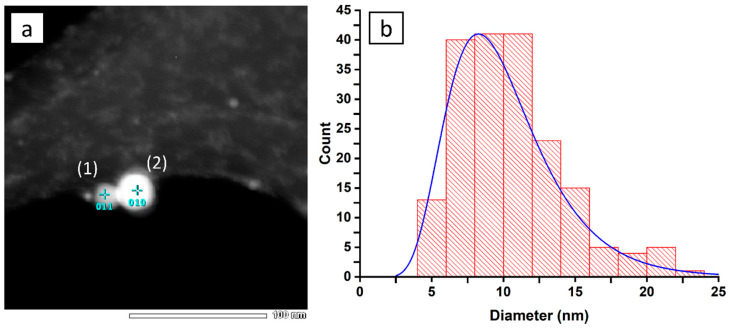
(**a**) TEM image of the nickel nanoparticles along with indicators showing EDX measurement sites, (**b**) histogram of the nanoparticle size distribution.

The TEM images from the dried nanoparticle colloid over the Cu grid reveal the size and shape of the obtained nanoparticles. Figure 3a shows that the nickel NPs are spherical, while Figure 3b presents their size distribution.

We can see that most of the nanoparticles detected can be included in the 10 ± 5 nm range. The EDX measurements (Table 1) give insight into the nanoparticle composition and indicate Ni:O ratio of ca. 54:1, which means that the particle is nearly purely metallic, while the detected oxygen is only residual.

This result lies in line with the UV-vis investigation, where only a faint band from oxidated nickel was detected. The difference in the NP diameter and hydrodynamic diameter provided by the DLS comes from a relatively large electric double layer, raising the measured hydrodynamic diameter of the nanoparticles.

**Figure 4:**
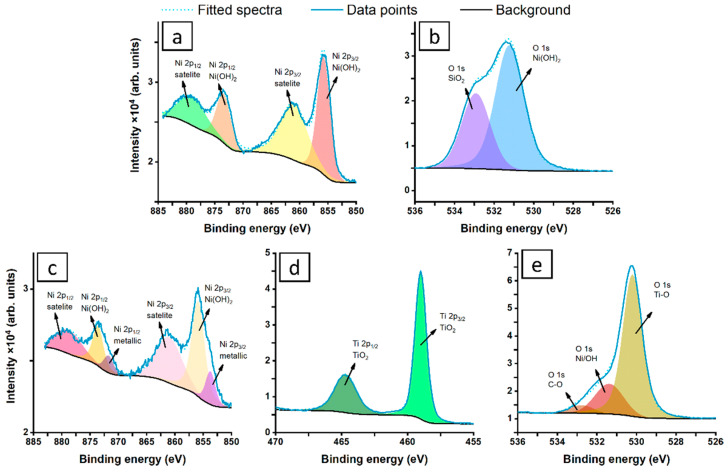
Deconvoluted XPS spectra for nickel nanoparticles placed on Si wafer (**a**,**b**), and onto titania nanotubes (**c**–**e**). Measurements were performed in the energy range of nickel (**a**,**c**), oxygen (**b**,**d**), and titanium (**e**).

The high-resolution X-ray photoelectron spectroscopy data were recorded to determine the chemistry of the NiNPs directly after laser ablation, and again after their deposition on the surface of the TiO_2_NTs. The composition of the NiNPs formed by laser ablation is straightforward, and the Ni2p spectra are presented in Figure 4.

A single peak doublet was used for the deconvolution model, with Ni2p_3/2_ peaking at 855.7 eV, which is characteristic for Ni(OH)_2_ forming a shell over the metallic core [57,58]. The Ni(OH)_2_ is characterized by significantly split spin-orbit components (by 17.7 eV) and strong satellite features, where Ni2p_3/2_ of Ni(OH)_2_ satellite peaks are located at 861 eV, as indicated in Figure 4a [59]. The deconvolution of the Ni2p spectra recorded for NPs placed on the TiO_2_NTs (Figure 4c), however, shows more complex surface chemistry, revealing an additional peak doublet. The Ni2p_3/2_ peak located at 853.7 eV belongs to NiO, with the Ni2p_3/2_ NiO satellite merging with Ni(OH)_2_ at approximately 861 eV [58,60]. Based on the proposed deconvolution, the share of the dehydrated NiO in the TiO_2_NTs decorated with NiNPs is around 20%. However, it should be noted that the observed change is not parallel with the modification of TiO_2_ chemistry, based on recorded Ti2p spectra (Figure 4d), with Ti2p_3/2_ peaking at 459.0 eV [61,62]. Finally, the O1s spectra for Ni colloid (Figure 4b) reveal the presence of multiple components; therefore, two spectral components were proposed for deconvolution. The primary signal peaking at 531.4 eV is characteristic for Ni(OH)_2_ species; however, its high share with respect to Ni2p peak is due to the presence of air-grown adventitious carbon contaminations [63,64]. The second, weaker O1s component is positively shifted at 1.7 eV and should be interpreted as SiO_2_ signal from the silicon substrate for the nanoparticles but also C–O species of air-grown contaminants [65,66]. Thus, this signal was excluded from further analysis. In Figure 4e, the O1s spectra dominated by strong TiO_2_ component (530.2 eV) can be seen. Full deconvolution data are shown in Table 2.

### 3.2. Electrode Morphology

**Figure 5:**
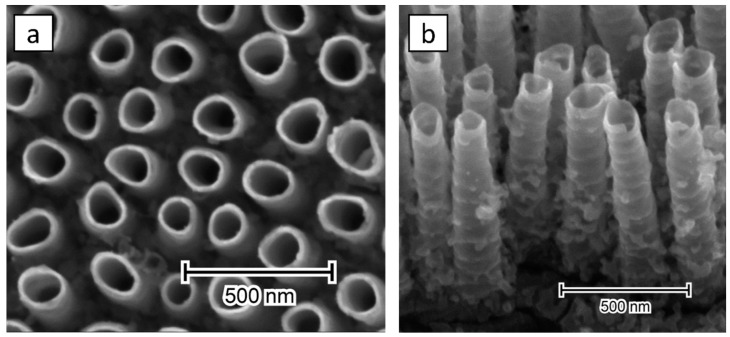
(**a**) Top and (**b**) side SEM image of the TiO_2_ nanotubes after ultrasonic-treatment in a nickel bath.

The images of the geometry, provided by the SEM, are shown in Figure 5.

The as-anodized nanotubes are about 800 nm long, have an inner diameter of 120 nm, a wall thickness of 11 nm, and are separated by 90 nm, which is consistent with our previous study [37]. Their overall morphology is not altered by ultrasonic bath modification either, aside from a few bald patches of detached NTs (Figure S1). Although titania platform has ultrasound-supported contact with the Ni-rich colloid, no NPs can be seen either over the rims nor along the tubular walls.

### 3.3. Electrochemical Performance

**Figure 6:**
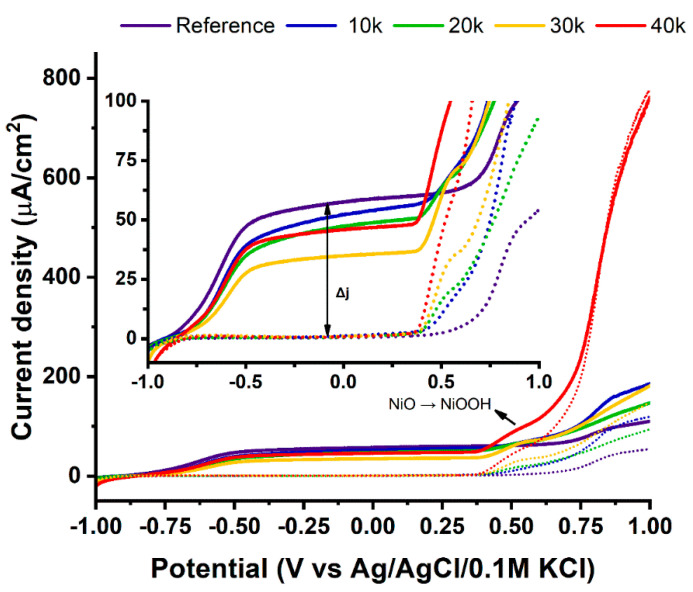
Linear voltammograms of the modified titania nanotubes investigated in the dark (dotted line) and under simulated AM 1.5 light (solid line).

The linear voltammetric sweeps (Figure 6) showcase how quick and simple ultrasound bath modification can influence material properties.

In the presented curves, the presence of nickel is apparent at +0.55 V where oxidation of NiO to NiOOH occurs [67], and passivation of the surface defects can be observed at ca. +0.9 V [68]. At the potentials below oxygen evolution reaction (OER), the reference sample has the highest current densities, which trend downwards with the amount of nickel deposited. The ultrasound-induced detachment of a small number of nanotubes might be responsible for the reduction in materials’ active surface area (Figure S1) [69]. This phenomenon is, however, heavily dependent on the geometry of the nanotubes used, as our group has previously shown the absence of any ultrasound-induced changes in densely-packed NTs [70]. On the other hand, the nickel nanoparticles may be responsible for higher recombination of light-generated charge carriers, which can also explain lower photocurrent values [71]. In the dark at +1.0 V, the reference sample reaches a current density of 56 μA/cm^2^ and twice as much under simulated solar radiation, which is not enough to produce meaningful amounts of gaseous product. Simultaneously, dipping the nanotubes in the nickel nanoparticle solution results in modified titania exhibiting much greater current densities compared to bare substrate, confirming that NiNPs provide titania platform with the activity toward efficient oxygen evolution. Ultrasound bath-coating with nanoparticles created by 40 thousand laser pulses raises the obtained currents over 13-fold, up to 780 μA/cm^2^ at +1.0 V vs. Ag/AgCl/0.1M KCl. It should be also pointed out that the electrode labelled as 40k being polarized in the anodic limit and exposed to radiation does not change its response regarding dark conditions, while for others the irradiation enhances recorded currents in the whole potential range. Such behavior is due to the fact that the oxygen evolution reaction (OER) dominates the total response and as a result the photoactivity is simply hidden [72]. Therefore, with a simple, quick, and easy step, the obtained current densities can be boosted by over an order of magnitude where, in case of structural optimizations [35], or doping [72], only a few percent increase is often observed.

**Figure 7:**
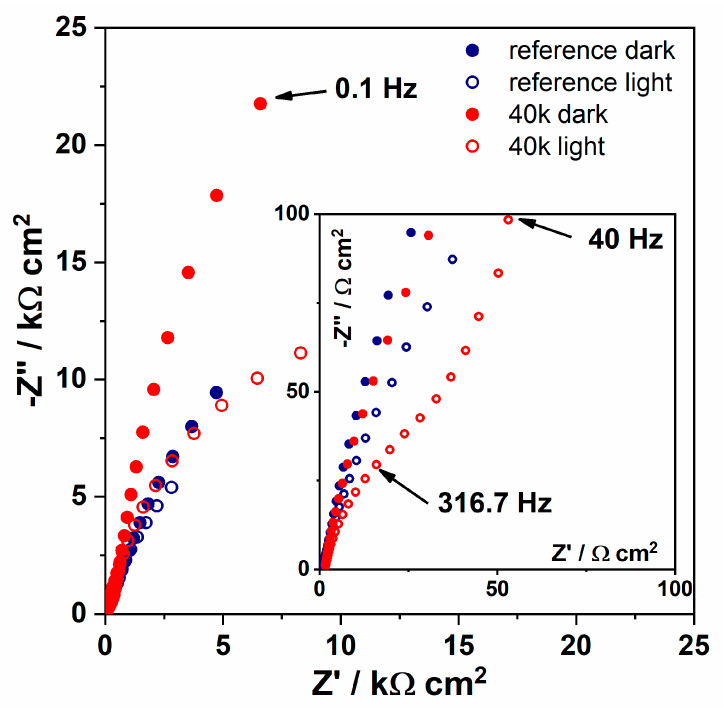
Nyquist representation of impedance spectra for Ni-decorated titania (40k) and reference material recorded in dark and under UV-vis irradiation.

In the Nyquist plots shown in Figure 7, the real and negative imaginary components of the complex impedance Z are plotted on the horizontal and vertical axis, respectively.

Regarding dark conditions, the impedance in the medium and high frequency regime is related to the internal resistance and charge transfer at the titania NTs/Ti substrate interface, respectively, while the highest frequency limit is related to the electrolyte resistance [73]. Since the measurements were performed in the same electrolyte and both electrodes (reference and Ni-decorated) are based on the substrate consisting of titanium plate overgrown by the ordered nanotubes, the similar shape of the spectra indicates that the presence of Ni nanoparticles does not change the internal resistance of TiO_2_NTs. In the lower frequency range, where the signal is attributed to the Nernstian diffusion within the electrolyte (mHz range) and the electron transport at the electrode/electrolyte impedance (1–100 Hz range), the dark impedance is higher for the modified material. It results from the reduced diffusion of charges within the material pores [74] due to the presence of the nanoparticles probably located mostly in the surface zone of titania. When both the materials were exposed to light the decrease in the impedance is observed in the whole frequency regime, suggesting that photogenerated electron-hole pairs lower the overall electrode resistance [75]. Nevertheless, the difference in the impedance spectra recorded in both conditions is much higher for 40k sample due to the facilitated charge transfer kinetics supported by incorporated nickel species.

## 4. Conclusions

The eco-friendly synthesis of the nickel nanoparticles via pulsed laser ablation in liquid was demonstrated. The UV-vis and EDX investigation revealed nearly pure, metallic form of nickel, with only a residual amount of oxygen included in the hydroxide surface species. The DLS study shows that laser processing has a homogenizing effect on the nanoparticles, while their size could be controlled by the management of the temperature of the solution during the process. Facile modification of the titania nanotubes was performed via only five-min long immersion in NiNPs suspension without any additional chemicals, further purification step or high-temperature annealing. According to EDX and XPS analysis, nanoparticles contain a metallic core covered with the hydroxide shell. Although the SEM inspection does not confirm the presence of nanoparticles embedded in the ordered titania system, the results of electrochemical investigations indicate significant change in the materials activity both in dark and under irradiation. The recorded impedance spectra reveals significant decrease in impedance for the modified titania irradiated by the solar light, suggesting efficient charge separation and collection at the back contact. Moreover, the incorporation of the nickel nanoparticles provides Faradaic activity attributed to the NiO to NiOOH redox reaction and over an order of magnitude boosted OER performance. The proposed approach, in which any complex metal precursor or organic surfactants are not utilized, can be regarded as a promising alternative to wet chemistry modification methods. We hope that the presented results will inspire researchers to take advantage of the laser-assisted routes used for the nanomaterials synthesis, as they can be eco-friendly, time and cost-efficient.

## Figures and Tables

**Table 1:** EDX data taken from two measurement sites indicated in Figure 3a.

Element	Energy (keV)	At._(1)_ %	At._(2)_ %
C	0.227	16.46	14.89
O	0.525	1.58	1.48
Ni	7.471	81.96	83.64

**Table 2:** Binding energies (BE) of core levels: Ni2p, Ti2p and O1s present for the Ni nanoparticles, and the chemical analysis (in at. %).

	Ni2*p*		Ti2*p*	O1*s*	
	NiO	Ni(OH)_2_	TiO_2_	Ti-O	Ni/OH
BE (eV)	853.7	855.7	459.0	530.2	531.4
NiNP (%)	--	17.8	--	--	82.2
NiNP@TiO_2_ (%)	1.0	3.9	24.9	54.4	15.8

## Supplementary Materials

The following are available online at https://www.mdpi.com/1996-1944/13/18/4068/s1, Figure S1: SEM images of the titania nanotubes indicating spots where the nanotubes detached during ultrasound treatment.
