# Supplementary material for: Laser-Assisted Synthesis and Oxygen Generation of Nickel Nanoparticles
Source: Materials (Basel). 2020 Sep 13;13(18):4068. doi: 10.3390/ma13184068 (PMC7560387; doi:10.3390/ma13184068)
Supplement: Supplementary file 1 [file materials-13-04068-s001.pdf]

materials

Article

Laser-Assisted Synthesis and Oxygen Generation of Nickel Nanoparticles

Jakub Wawrzyniak [1,*], Jakub Karczewski [2], Jacek Ryl [3], Katarzyna Grochowska [1] and Katarzyna Siuzdak [1]

[1] Centre for Plasma and Laser Engineering, The Szewalski Institute of Fluid-Flow Machinery, Polish Academy of Sciences, Fiszera 14 st., 80-231 Gdańsk, Poland; kgrochowska@imp.gda.pl (K.G.); ksiuzdak@imp.gda.pl (K.S.)
[2] Faculty of Applied Physics and Mathematics, Gdańsk University of Technology, Gabriela Narutowicza 11/12 st., 80-233 Gdańsk, Poland; jakub.karczewski@pg.edu.pl
[3] Faculty of Chemistry, Gdańsk University of Technology, Gabriela Narutowicza 11/12 st., 80-233 Gdańsk, Poland; jacek.ryl@pg.edu.pl
* Correspondence: jwawrzyniak@imp.gda.pl

Received: 12 August 2020; Accepted: 11 September 2020; Published: 13 September 2020

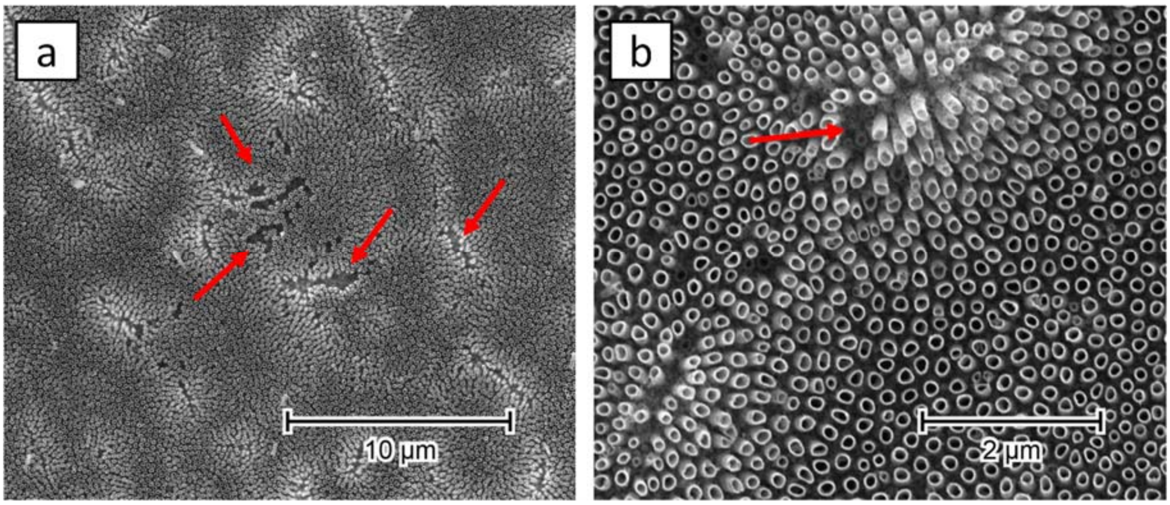

Figure S1. SEM images of the titania nanotubes indicating spots where groups of nanotubes detached during ultrasound treatment (**a**) and spots where only single nanotubes were removed (**b**).
